# Isolated muscle metastasis from early-stage endometrial carcinoma six years after diagnosis: A case report and literature review

**DOI:** 10.1016/j.ijscr.2025.111032

**Published:** 2025-02-07

**Authors:** Faten Limaiem, Aziz Atallah, Hafedh Mestiri

**Affiliations:** a University of Tunis El Manar, Faculty of Medicine of Tunis, 1007, Tunisia; b Pathology Department, Hospital Mongi Slim, La Marsa, Tunisia; c Department of Visceral Surgery, Hospital Mongi Slim, La Marsa, Tunisia

**Keywords:** Endometrial cancer, Early stage, Muscle metastasis, Pathology, Surgery

## Abstract

**Introduction and importance:**

Endometrial cancer is the most common gynecological malignancy, typically diagnosed at an early stage with a favorable prognosis. However, certain subtypes and molecular characteristics can predispose patients to a higher risk of recurrence and metastasis. Understanding unusual metastatic patterns, such as isolated muscular metastases, is crucial for comprehensive management and treatment strategies in endometrial cancer.

**Case presentation:**

A 55-year-old woman with a previous diagnosis of grade 2 intra-mucosal endometrioid adenocarcinoma presented with a painful abdominal swelling. Imaging identified a parietal formation within the rectus abdominis muscle, initially suspected to be an endometriotic cyst. Subsequent surgical excision confirmed the presence of carcinomatous glands infiltrating the striated muscle tissue. After a complication-free surgery, the patient underwent adjuvant chemotherapy and is now being monitored for 10 months without any signs of recurrence.

**Clinical discussion:**

The presented case underscores the rarity of muscular metastases in endometrial cancer, emphasizing the need for better understanding and awareness of unusual metastatic patterns. Detailed histological examination confirmed the secondary parietal muscular localization of the carcinoma, prompting further therapeutic management.

**Conclusions:**

This case contributes to the existing literature by highlighting a unique presentation of metastatic endometrial cancer in the muscular tissue, shedding light on potential diagnostic challenges and treatment considerations. Enhanced awareness and knowledge of such atypical metastatic sites are crucial for improved patient care and outcomes.

## Highlights

• Metastases of endometrial carcinoma to the musculoskeletal system are infrequent.
• There is limited available data on the optimal management of metastatic disease in the musculoskeletal system
• Factors such as tumor size, specific histopathological and molecular features, and certain tumor subtypes are linked to poorer prognosis and increased risk of relapse.
• Individualized treatment is crucial, with surgical resection of isolated metastasis and adjuvant therapy considered for certain cases with favorable performance statuses.
• A multidisciplinary approach is crucial in treating rare isolated muscular metastasis in endometrial cancer effectively, ensuring comprehensive patient care.

## 1 Introduction

Metastatic disease in endometrial cancer, although uncommon, poses a significant risk for certain patients despite the overall low rates of relapse. Factors such as tumor size, histopathological features, and specific molecular characteristics can notably heighten this risk [[1], [2], [3]]. Endometrial cancer typically metastasizes to sites such as the cervix, parametria, pelvic and paraaortic lymph nodes, with less common occurrences in the lungs, liver, bones, and brain. Isolated muscle metastases are infrequently documented [1,2], underlining a crucial gap in the current understanding of metastatic patterns in endometrial cancer, particularly concerning muscular metastases. This study aims to address this gap by presenting a case of isolated muscle metastasis originating from early-stage low-grade endometrial carcinoma, emerging six years post the initial diagnosis. Through this case study, our goal is to contribute to the existing literature and enhance awareness regarding the potential for unusual metastatic presentations in endometrial cancer.

This case report has been presented in accordance with the SCARE Criteria [4].

## 2 Case presentation

### 2.1 Patient history and presenting complaint

A 55-year-old woman, with an otherwise unremarkable medical history and no current medications, underwent a total hysterectomy and bilateral adnexectomy in 2018 for grade 2 intra-mucosal endometrioid adenocarcinoma. The tumor was confined to the endometrium, showing signs of lymphovascular emboli (<5 emboli) but no perineural invasion or lymph node metastasis. According to the FIGO staging criteria, it was categorized as stage IA. Recently, she presented to a general practitioner for evaluation due to a progressively painful abdominal swelling below the umbilicus, which had emerged over the past three months.

### 2.2 Physical examination findings

On physical examination, the patient appeared in overall good health, classified as WHO 1 and ASA 1, and was afebrile. The abdomino-pelvic exam revealed a firm, stony mass approximately 7 cm in size, located below the umbilicus and lateralized to the right. The mass was fixed to deeper structures, mildly tender on palpation, and lacked signs of local inflammation.

### 2.3 Diagnostic workup

**Fig. 1:**
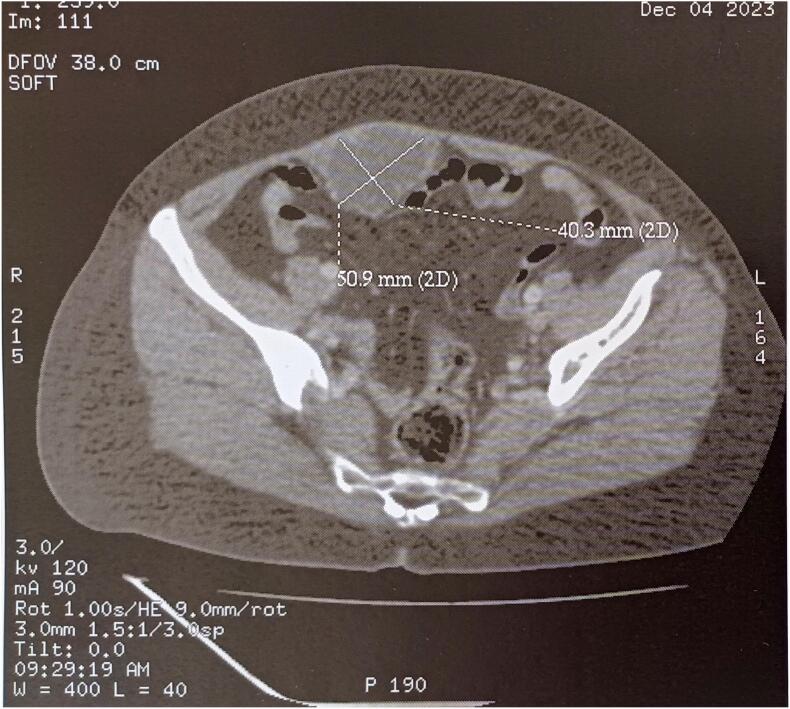
Sagittal CT scan depicted a solid-cystic parietal formation within the rectus abdominis muscle, situated below the umbilicus, featuring a budding tissue portion measuring 50 × 40 × 70 mm.

**Fig. 2:**
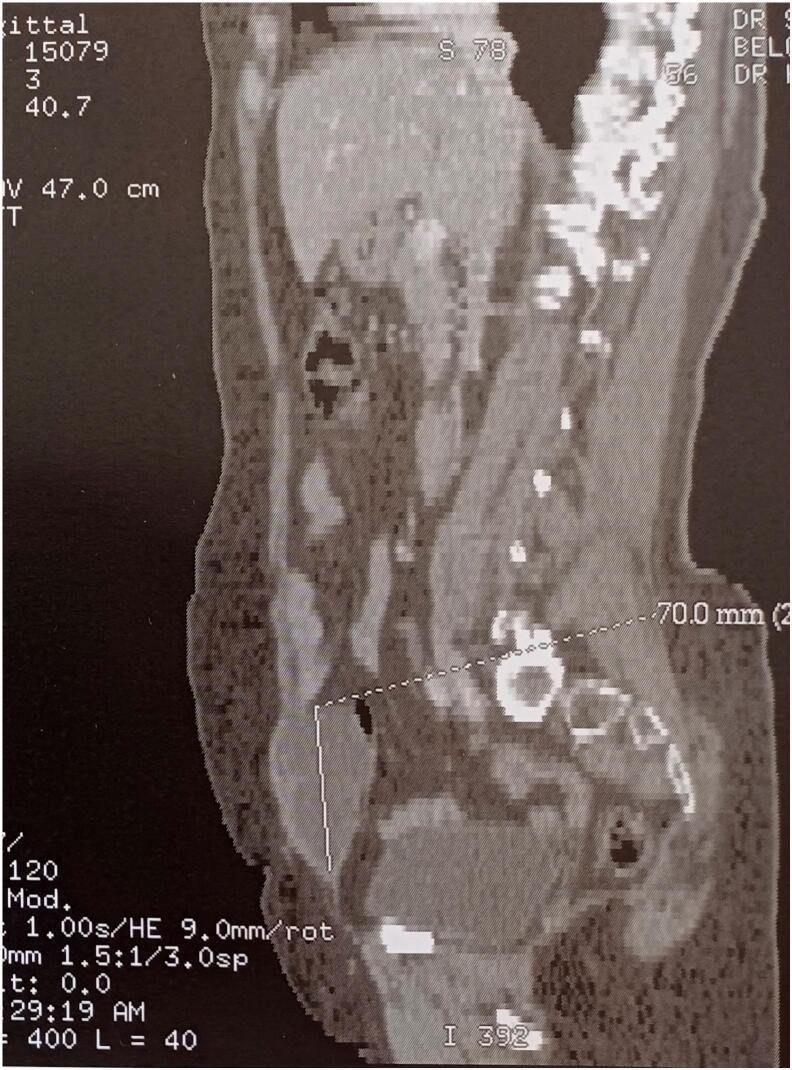
Axial CT scan displayed a solid-cystic parietal formation within the rectus abdominis muscle, positioned below the umbilicus, with a budding tissue portion measuring 50 × 40 × 70 mm.

Abdominal-pelvic CT imaging demonstrated a solid-cystic mass within the rectus abdominis muscle, located below the umbilicus, with a budding tissue component measuring 50 × 40 × 70 mm (Fig. 1, Fig. 2). This finding raised suspicion for either a desmoid tumor or an endometriotic cyst. Abdominal-pelvic MRI further revealed an extensive, primarily cystic lesion, initially thought to be an endometriotic cyst, affecting the abdominal wall and involving the rectus abdominis muscle. The lesion exhibited thin septations, with a suspicious enhancing nodule in the mid-third of the anterolateral aspect, which demonstrated early enhancement following gadolinium injection and delayed washout. Significant diffusion restriction was noted on ADC mapping.

### 2.4 Surgery

The surgical approach entailed a meticulous wide local excision of the solid-cystic mass, with a primary focus on achieving negative margins. An open surgical technique was utilized due to the mass's adherence to deeper anatomical structures, which necessitated a more extensive dissection. Throughout the procedure, careful and deliberate dissection was performed to prevent any rupture of the mass, thereby minimizing the risk of spillage and potential complications. Following the excision, the resected mass was promptly sent to the pathology department for thorough evaluation and diagnosis.

### 2.5 Pathology findings

**Fig. 3:**
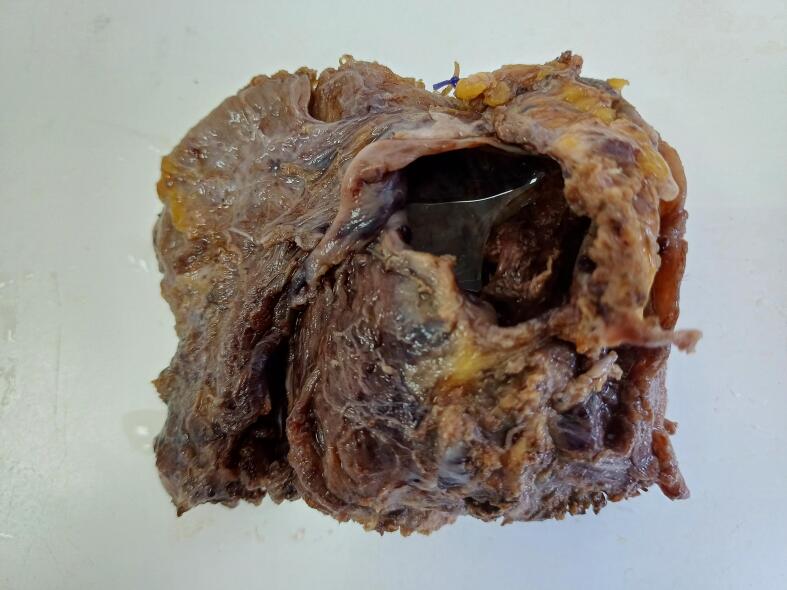
Macroscopic examination of the surgical specimen unveiled a brownish solid and cystic mass measuring 7 × 5 × 4 cm.

**Fig. 4:**
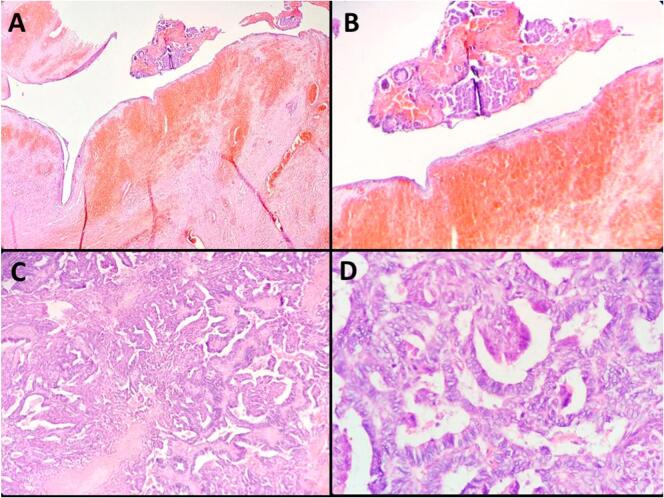
A: Histological analysis of the surgical resection specimen revealed a cystic portion with a fibrous wall punctuated by hemorrhagic infiltrations. Inside the lumen, hemorrhage was observed alongside carcinomatous glands. (Hematoxylin and eosin, magnification × 100). B: Histologically, the cystic component of the resected mass is composed of a fibrous stroma interrupted by hemorrhagic suffusions. The cyst lumen contains carcinomatous glands as well as hemorrhage. (Hematoxylin and eosin, magnification × 400). C: Histological analysis revealed the solid component of the excised mass to consist of fused, intricate back-to-back glandular structures. (Hematoxylin and eosin, magnification × 100). D: The tumor cells exhibited a cylindrical shape, with eosinophilic cytoplasm and moderately atypical ovoid nuclei that showed mitotic activity. (Hematoxylin and eosin, magnification × 400).

Upon gross examination, the excised mass appeared brownish in color and presented a combination of solid and cystic components with a firm consistency (refer to Fig. 3). It weighed 53 g and measured 7 × 5 × 4 cm. A histological analysis unveiled that the cystic portion featured a fibrous wall with hemorrhagic infiltration, housing hemorrhage and carcinomatous glands (see Figs. 4 and B). The solid component comprised merged complex glandular structures devoid of intervening stroma (depicted in Fig. 4C). Tumor cells displayed cylindrical morphology with eosinophilic cytoplasm and moderately atypical ovoid nuclei, exhibiting mitotic activity (as shown in Fig. 4D). Carcinomatous cells infiltrated the striated muscle tissue at the periphery. Immunohistochemical analysis indicated a positive expression of CK7, estrogen receptor, progesterone receptor, and mismatch repair proteins (MLH1, MSH2, MSH6, and PMS2) in the endometrial glandular epithelial cells. Furthermore, a mutant expression pattern of P53 was observed, with over 80 % positivity in these cells. CK20 staining was negative.

### 2.6 Postoperative course and follow-up

The patient's postoperative recovery progressed smoothly, marked by stable vital signs and the absence of complications. Subsequently, the patient was promptly referred to the oncology department for comprehensive management. This included the initiation of adjuvant chemotherapy as part of a personalized treatment plan for metastatic endometrial cancer. The standard adjuvant approach in this case involved administering a carboplatin/paclitaxel combination over 4 cycles. Throughout the treatment phase, the patient received regular monitoring, including CT imaging every 3 months, routine toxicity assessments, and evaluations of quality of life. The follow-up protocol specified for this patient includes physical examinations every 3 months over the next 2 years, CT imaging every 3–6 months during this period, and annual imaging thereafter if the patient remains disease-free. Currently, the patient remains under close monitoring, with a follow-up period extending to 10 months, during which no signs of recurrence have been identified.

## 3 Discussion

Endometrial carcinoma ranks as the sixth most prevalent cancer among women globally and stands as the primary gynecologic malignancy in Europe and North America [3]. The age-standardized incidence rates were recorded at 20.2 and 21.1 per 100,000 in 2020, respectively [3]. Endometrial carcinoma is classified into various histological categories according to cell type. The most prevalent type is endometrioid, representing 75–80 % of cases, and further categorized from grade 1 (well differentiated) to grade 3 (poorly differentiated) [5,6]. In the 5th edition of the WHO Classification of Female Genital Tumors, four distinct molecular subtypes of endometrial carcinoma are now recognized: POLE-ultramutated, mismatch repair-deficient (MMRd), p53-mutant, and tumors without a specific molecular profile. The 2020 WHO classification underscores the importance of prioritizing POLE mutations as a key diagnostic criterion over other molecular abnormalities [7,8]. Endometrial carcinomas with POLE mutations are associated with an excellent prognosis, particularly in Grade 3 endometrioid adenocarcinomas [7,8]. However, the identification of this subgroup remains challenging, as there are no specific immunohistochemical markers for POLE mutations. On the other hand, MMRd endometrioid carcinomas exhibit high microsatellite instability and accumulate mutations at a rapid rate. These tumors are typically identified through immunohistochemistry for mismatch repair (MMR)-related proteins [7,8]. The p53-mutant subgroup, characterized by frequent TP53 mutations and typically associated with serous histology, corresponds to the Type II tumors in endometrial carcinoma. A fourth subgroup, comprising tumors without a specific molecular profile, is the most common in The Cancer Genome Atlas dataset. These tumors exhibit no significant mutations or copy number variations, and while they share some characteristics with the MMRd subgroup, they generally present with an intermediate prognosis [7,8]. In line with these molecular advances, the 2020 WHO classification was followed by the introduction of a new staging system by FIGO in 2023. This updated system places greater emphasis on diagnostic factors such as histological type, grade, lymphovascular space invasion, and molecular alterations [7,8]. The vast majority of Stage I endometrial cancer cases without adverse prognostic indicators exhibit a 5-year overall survival rate exceeding 90 % [5,9]. Nonetheless, about 11 % of individuals with endometrial cancer experience disease recurrence [5,10]. The pattern of recurrent disease shows diversity, as certain research indicates that about half of patients experience local recurrence, while others suggest that most recurrences are distant or multifocal [5,11]. A minority of women may encounter an isolated central pelvic recurrence [5,11]. Endometrial carcinoma can disseminate through various pathways, influenced by the histological type and local invasion. The spread patterns may involve direct extension, lymphatic and hematogenous dissemination, as well as the retrograde passage of neoplastic cells through the fallopian tubes [6]. Certain factors such as tumor size and specific histopathological and molecular features like TP53abn and L1CAM positivity, along with tumor subtypes such as clear cell, serous, undifferentiated, mixed (>10 %), and carcinosarcomas, are linked to a poorer prognosis and an increased risk of relapse [3]. Recurrences of endometrial cancer commonly manifest in the vaginal cuff, pelvic and para-aortic lymph nodes, peritoneum, lungs, and liver. Uncommon sites of recurrence encompass the abdominal wall and muscles (2–6 %), spleen (1 %), central nervous system (<1 %), extra-abdominal lymph nodes (0.4–1 %), and, less frequently, the adrenals, pancreas, and appendix [12]. In case reports published over the last decade, the majority of musculoskeletal metastases have been documented in the context of widespread metastatic disease, with isolated metastases being a rare occurrence. To the best of our knowledge, only four cases of isolated muscular metastasis of endometrial carcinoma have been documented in the literature (refer to Table 1) [1,3,5,13]. Abdominal wall metastases have been associated with surgical incisions, irrespective of the surgical method employed (laparotomy or laparoscopy). While uncommon, such occurrences have been documented in cases of endometrial carcinoma [14]. The precise mechanism behind this phenomenon is often attributed to hematogenous dissemination to the site of recent trauma, the seeding of cancer cells following direct interaction between the tumor and the wound, the impact of pneumoperitoneum, surgical techniques, and local immune responses [12]. The management of port-site metastases and recurrences at laparotomy wounds involves a thorough investigation to exclude other metastatic sites. If distant disease is not detected, comprehensive excision and exploratory laparotomy or laparoscopy should be considered [14].

**Table 1:** Cases of muscle metastasis from endometrial carcinoma reported in the literature.

Author	Year	Age	Histology grade	Initial treatment	Site of muscle metastasis	Initial stage of endometrial cancer	Metastasis onset interval	Treatment of recurrence
Djurdjević S et al. [13]	2006	NP*	Endometrioid adenocarcinoma Grade 1	Surgery, chemotherapy, &Brachytherapy	Rectus abdominis and the psoas muscles	Stage IIIA	NP*	Surgery 4 cycles of paclitaxel and adjuvant irradiation
Oaknin A et al. [5]	2010	69	Endometrioid adenocarcinoma Grade 1	Total abdominal hysterectomy and bilateral salpingo-oophorectomy.	Deltoid Muscle	Stage IA (FIGO)	7 years	Concurrent chemoradiotherapy
Hayek J et al. [1]	2021	73	Endometrioid Adenocarcinoma Grade 2	Surgery Vaginal cuff brachytherapy	Psoas Muscle	Stage II (FIGO)	11 months	Six cycles of IV carboplatin and paclitaxel Pelvis radiation
Heidinger M [3]	2022	83	Endometrioid Adenocarcinoma Grade 3	Surgery, adjuvant chemotherapy and vaginal brachytherapy but declined external beam radiotherapy	Quadriceps femoris	Stage IIIC2 (FIGO)	15 months	Concomitant palliative chemotherapy and external beam radiotherapy to the right femur.
Our case	2024	55	Endometrioid Adenocarcinoma Grade 2	Total hysterectomy, bilateral adnexectomy	Rectus Abdominis Muscle	Stage IA (FIGO)	6 years	Surgery and Adjuvant chemotherapy

NP: not precised.

Individualized treatment is essential, with radical surgical resection of isolated metastasis being a viable consideration for patients with a good performance status, provided that PET-CT scans have excluded other distant metastatic sites [14]. While many treatment guidelines advocate for surgical resection and adjuvant therapy in specific cases with favorable performance statuses, this approach can potentially enhance survival rates [14]. However, the overall prognosis is often poor, leading to palliative care emerging as the primary treatment option in certain instances. In cases of unresectable or disseminated metastases, chemotherapy becomes a viable option. Combination therapies involving paclitaxel and carboplatin or cisplatin are commonly prescribed for recurrent endometrial cancer [14].

## 4 Conclusion

In summary, this case underscores a rare instance of a single metastatic site of endometrioid adenocarcinoma on the rectus abdominis muscle without other signs of advanced disease. Additionally, the detailed exploration of this case report emphasizes the significance of recognizing and investigating uncommon metastatic presentations in cancer, particularly isolated muscle metastases in endometrial cancer. The insights provided from the patient's journey, diagnostic process, treatment, and follow-up contribute valuable information to the understanding of these unusual metastatic patterns. This case serves as a reminder for clinicians to remain vigilant for rare metastatic sites, even in early-stage cancers, and highlights the importance of prompt identification and multidisciplinary management in addressing such cases effectively. Further research and ongoing vigilance are crucial for improving our knowledge of metastatic behaviors in endometrial cancer and refining therapeutic approaches for similar uncommon presentations in the future.

## Abbreviations

ADC: Apparent Diffusion Coefficient
ASA: American Society of Anesthesiologists Physical Status Classification
CT scan: Computed Tomography Scan
FIGO: Federation of International Gynecology and Obstetrics
MRI: Magnetic Resonance Imaging
MSH2: MutS Homolog 2
MSH6: MutS Homolog 6
MMR: Mismatch Repair
MMRd: Mismatch Repair Deficient
PET-CT scans: Positron Emission Tomography – Computed Tomography Scan
PMS2: Postmeiotic Segregation Increased 2
POLE: Polymerase epsilon
WHO: World Health Organization

## Author contribution

Dr. Faten LIMAIEM: Prepared, organized, wrote, and edited all aspects of the manuscript.

Dr. Aziz ATALLAH, and Pr Hafedh MESTIRI: Read, edited, and approved the final version of the manuscript. Contributed to data acquisition, analysis, and interpretation. Provided final approval of the manuscript before its submission.

## Consent

Written informed consent was obtained from the patient for publication of this case report and accompanying images. A copy of the written consent is available for review by the Editor-in-Chief of this journal on request.

## Ethical approval

Ethical approval for this study was provided by the Ethical Committee of Mongi Slim University Hospital, Marsa, Tunisia.

## Guarantor

Dr. Faten Limaiem

## Provenance and peer review

Not commissioned, externally peer-reviewed.

## Funding

This research did not receive any specific grant from funding agencies in the public, commercial, or not-for-profit sectors.

## Declaration of competing interest

None declared.
